# Toward Unsupervised Capacity Assessments for Gait in Neurorehabilitation: Validation Study

**DOI:** 10.2196/66123

**Published:** 2025-03-26

**Authors:** Aileen C Naef, Guichande Duarte, Saskia Neumann, Migjen Shala, Meret Branscheidt, Chris Easthope Awai

**Affiliations:** 1 Data Analytics & Rehabilitation Technology (DART) Lake Lucerne Institute Vitznau Switzerland; 2 Rehabilitation Engineering Laboratory Department of Health Sciences and Technology ETH Zurich Zurich Switzerland; 3 Department of Neurology University Hospital of Zurich Zurich Switzerland; 4 cereneo, Center for Neurology and Rehabilitation Weggis Switzerland; 5 Department of Health Sciences and Technology ETH Zurich Zurich Switzerland

**Keywords:** gait analysis, gait rehabilitation, 10-meter walk test, stroke, unsupervised assessments, supervised assessments, sensors, motivation, capacity, monitoring, wearables, stroke survivors, quality of life

## Abstract

**Background:**

Gait impairments are common in stroke survivors, negatively impacting their overall quality of life. Therefore, gait rehabilitation is often targeted during in-clinic rehabilitation. While standardized assessments are available for inpatient evaluation, the literature often reports variable results when these assessments are conducted in a home environment. Several factors, such as the presence of an observer, the environment itself, or the technology used, may contribute to these differing results. Therefore, it is relevant to establish unsupervised capacity assessments for both in-clinic use and across the continuum of care.

**Objective:**

This study aimed to investigate the effect of supervision on the outcomes of a sensor-based 10-meter walk test conducted in a clinical setting, maintaining a controlled environment and setup.

**Methods:**

In total, 21 stroke survivors (10 female, 11 male; age: mean 63.9, SD 15.5 years) were assigned alternately to one of two data collection sequences and tested over 4 consecutive days, alternating between supervised test (ST) and unsupervised test (UST) assessments. For both assessments, participants were required to walk a set distance of 10 meters as fast as possible while data were collected using a single wearable sensor (Physilog 5) attached to each shoe. After each walking assessment, the participants completed the Intrinsic Motivation Inventory. Statistical analyses were conducted to examine the mean speed, stride length, and cadence, across repeated measurements and between assessment conditions.

**Results:**

The intraclass correlation coefficient indicated good to excellent reliability for speed (ST: κ=0.93, *P*<.001; UST: κ=0.93, *P<*.001), stride length (ST: κ=0.92, *P<*.001; UST: κ=0.88, *P<*.001), and cadence (ST: κ=0.91, *P<*.001; UST: κ=0.95, *P<*.001) across repeated measurements for both ST and UST assessments. There was no significant effect of testing order (ie, sequence A vs B). Comparing ST and UST, there were no significant differences in speed (*t*_39_=–0.735, *P=*.47, 95% CI 0.06-0.03), stride length (*z=*0.835, *P=*.80), or cadence (*t*_39_=–0.501, *P=*.62, 95% CI 3.38-2.04) between the 2 assessments. The overall motivation did not show any significant differences between the ST and UST conditions (*P*>.05). However, the self-reported perceived competence increased during the unsupervised assessment from the first to the second measurement.

**Conclusions:**

Unsupervised gait capacity assessments offer a reliable alternative to supervised assessments in a clinical environment, showing comparable results for gait speed, stride length, and cadence, with no differences in overall motivation between the two. Future work should build upon these findings to extend unsupervised assessment of both capacity and performance in home environments. Such assessments could allow improved and more specific tracking of rehabilitation progress across the continuum of care.

## Introduction

More than 80% of stroke survivors experience gait impairments, and between 55% and 75% continue to experience functional problems 3 to 6 months after their stroke [1,2]. These impairments frequently lead to reduced activity levels and diminished community participation [3-5]. Due to this, one of the most common goals in gait rehabilitation is to enhance functional independence and quality of life [3,6-8]. Despite these efforts, many survivors continue to experience gait deficits even after discharge from their inpatient rehabilitation programs.

Within the clinical setting, observational gait analysis and scaled assessments are often used to identify gait deficits and functional improvements [9,10]. In these assessments, visual observation is used to qualitatively identify gait deviations. These types of assessments are popular due to their simplicity and availability. However, the literature indicates that gait and mobility parameters differ when assessments are conducted at home versus in the clinical setting; this difference may become more relevant as advancements in digital therapeutics and telerehabilitation offer promising avenues for long-term rehabilitation outside the clinic [9-14]. These differences in assessment outcomes could stem from various factors, such as the transition from a supervised to an unsupervised setting, the change in the environment itself, or technical limitations [11]. Therefore, to determine the feasibility of conducting accurate and consistent assessments in a home environment, it is essential to first isolate and address these variables in a controlled manner using a standardized clinical assessment.

Among the array of tools used in the clinical setting to evaluate walking capacity is the 10-Meter Walk Test (10-MWT) [15-18]. The test measures walking speed in meters per second over a distance of 10 meters, with the speed being indicative of functional outcomes and prognosis for stroke survivors [19-21]. In addition to providing a strong indicator of functional ability and being a predictor of health outcomes, the 10-MWT is a particularly valuable tool due to its applicability across populations, its convergent validity with other clinical gait capacity assessments, and the fact that it is standardized and easy to administer [22,23]. Moreover, combining the 10-MWT with wearable sensors, such as inertial measurement units, allows for the extraction of additional spatiotemporal gait parameters. These parameters are not only robust, but they enhance the interpretation of clinical assessment outcomes and aid in detecting motor recovery poststroke as well as predicting prognosis after stroke [24-26]. Future precision rehabilitation approaches will rely more strongly on the assessment of these kinds of granular gait metrics, making it relevant to validate these in current systems [27,28]. Standardized assessments to measure capacity, such as the 10-MWT may be complemented by data from continuous monitoring, which measures performance [29,30].

While unsupervised assessments and continuous monitoring are mostly associated with home settings, there are also many useful applications within a clinical environment. First, unsupervised assessments can reduce the assessment load on clinical staff, thus enhancing clinical efficiency [31]. Second, unsupervised assessments can be performed more frequently due to their independence from clinical planning, which allows insights into day-to-day fluctuations of test outcomes [11]. Third, enabling patients to perform their own assessments in an unsupervised manner can mitigate observer effects on outcomes [32]. Fourth, giving patients some level of autonomy concerning some aspects of their rehabilitation process can empower them and increase their involvement and ownership [33]. Finally, exposing patients to unsupervised assessments within a controlled, in-clinic context already prepares them to continue these across the home aspect of the continuum of care, removing learning effects and boosting adherence [34]. In synthesis, introducing unsupervised capacity assessments within the clinical setting has potential benefits on multiple levels, while also laying the foundation for assessments across the complete continuum of care.

For these reasons, this study investigates the supervision aspect of capacity assessments by maintaining a controlled clinical environment and using a simple-to-use tool that can easily be transferred outside the clinic. In other words, this study will determine whether a standardized gait capacity assessment conducted in the clinical setting without supervision maintains the same validity, replicability, and motivation as when conducted with supervision. We hypothesize that the unsupervised assessments will provide results that are as reliable and replicable as those collected in a supervised manner with no differences in motivation. This study will provide insights into the self-administration of gait capacity assessments, providing a basis for interpreting any potential differences that may arise in future studies expanding into home or less controlled environments.

## Methods

### Participants and Setting

A convenience sample of 21 patients with stroke (10 female and 11 male), aged 40-89 (mean 63.91, SD 15.54) years, were enrolled in this study at the Swiss Neurorehabilitation Clinic “cereneo” (Lucerne, Switzerland). Enrollment took place between November 2021 and June 2023 and followed a cross-over design. All stroke survivors who were more than 1 month post stroke, aged 18 years or older, able to use a tablet, with a Berg Balance Score greater than 31, and the ability to walk for at least 2 minutes, met the inclusion criteria to participate in the study. Exclusion criteria were the presence of comorbidities potentially affecting gait (eg, polyneuropathy, orthopedic impairments, and orthostatic hypotension), uncorrected visual impairments, psychiatric comorbidities that may affect the participants’ ability to follow study protocols or other medical reasons that would hinder participation.

### Experimental Procedure

**Figure 1:**
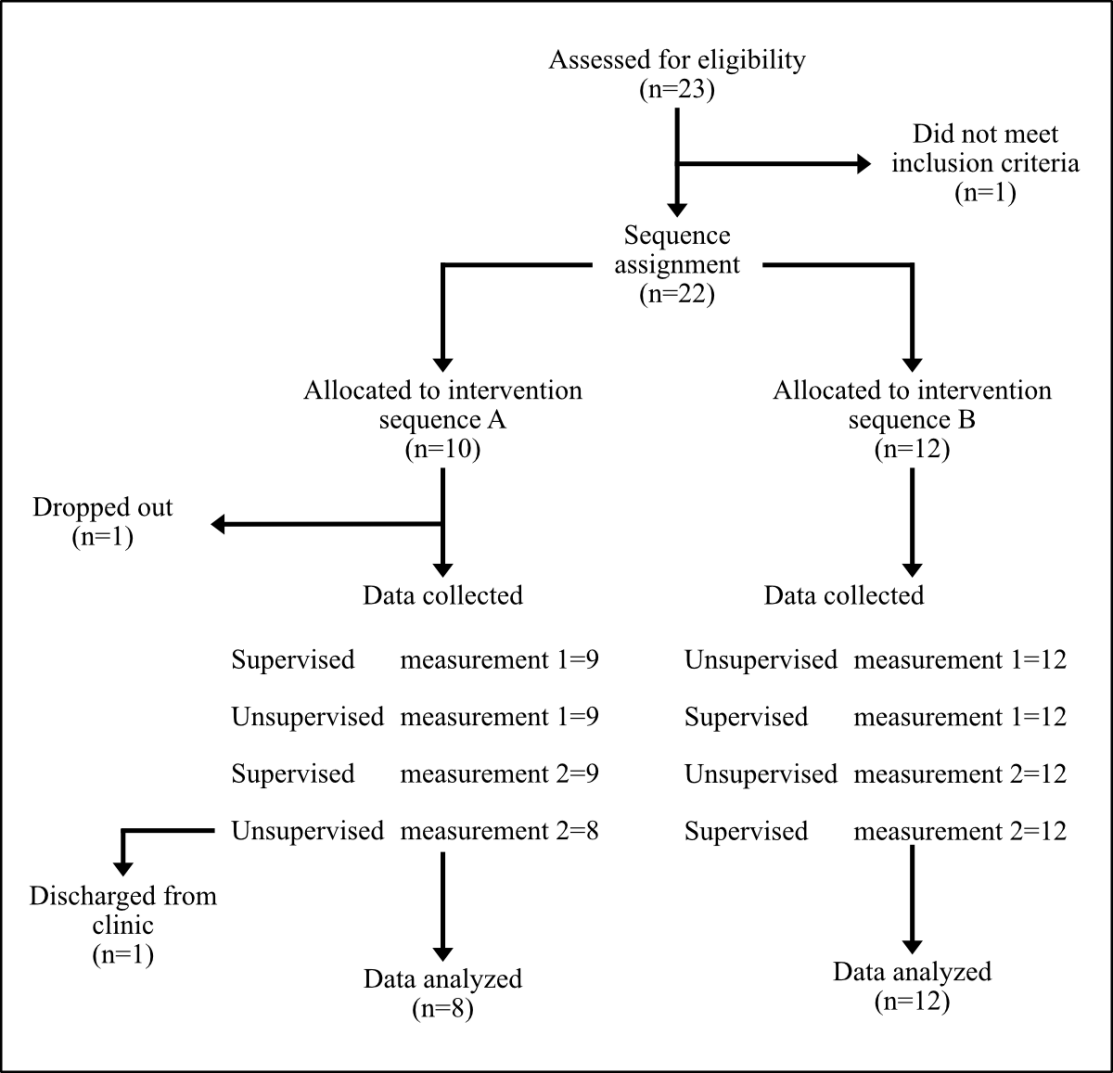
Schematic diagram outlining the enrollment and allocation to the intervention sequences of the study.

**Figure 2:**
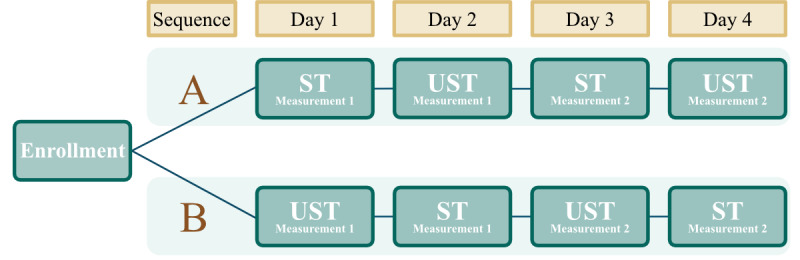
Study groups showing how participants were allocated to either sequence A or sequence B upon enrollment. Participants in both sequences completed the 10-MWT twice while supervised and twice while unsupervised. 10-MWT: 10-Meter Walk Test; ST: supervised test; UST: unsupervised test.

The total experimental procedure for this study was conducted across 4 days, with a single testing session per day. Upon enrollment in the study, participants were sequentially assigned in an alternating fashion to one of 2 sequences (A or B) representing differing protocol sequences (Figure 1). This approach was chosen to ensure balanced group sizes and facilitate study logistics. While this method does not introduce selection bias in individual assignments, it may not fully control for potential confounding factors in group distribution. Sequence A began with the 10-MWT conducted under supervised test (ST) conditions, while Sequence B began with the 10-MWT conducted under unsupervised test (UST) conditions (Figure 2). Participants then alternated the assessment type over the remaining 3 days (Figure 2). Regardless of the sequence to which they were assigned, participants always completed both the ST and the UST twice during the 4 days.

The goal of the 10-MWT was to have participants walk a set distance of 10 m as fast as possible in order to evaluate gait capacity. Markers at the 0- and 10-m points delineated the walking path to ensure consistent data collection conditions. To objectively evaluate the outcome, participants completed the assessment while wearing an inertial measurement unit on each shoe.

For all STs, the therapist prepared the participant by attaching the inertial measurement units to the shoes and positioning them at the start line. The therapist then counted down the start, and the participant walked as fast as possible over the marked finish line. The therapist was responsible for starting the stopwatch using the tablet app and stopping it when the finish line was reached. The therapist was present for the entire setup and data collection but did not provide feedback or guidance.

The UST was conducted following the same procedure as the ST. However, there was no direct supervision of the tasks. During the UST, a therapist or nurse was available in the adjacent nursing room for technical assistance and support during the setup. The participants were required to go to this room and pick up the tablet and sensors before walking to the designated track. If participants were unable to attach the inertial measurement units to the shoes themselves, the nurses or therapists attached them in the nurses’ office. Additionally, if the participants were unable to hold the tablet, for example, due to hemiparesis, the tablet was attached to the participant’s arm via a strap so that they could operate it with one hand. Once at the starting line, the participants got in place and started the stopwatch using the tablet app before walking as fast as they could over the finish line. Once across the finish line, the participants stopped the time using the same stopwatch app. The participants then removed the sensors and returned them, together with the tablet, to the nursing office. During the UST, the only interaction with the therapist was to attach or remove the sensors in the nursing office if the participant could not do it alone.

In both conditions, participants were required to complete the Intrinsic Motivation Inventory on paper in the nurses’ office. The participants were required to do this on their own with no help or interaction with the nurses or therapists. Participants in both sequences also always had a written information sheet available to them in both the ST and UST conditions, listing the required steps and acting as a user guide for the tablet app. In both conditions, there was no interaction between the participants and nurses or therapists, the only difference was that during the ST task, the nurse or therapist was there to start and stop the stopwatch and observe the data collection. If there were any problems, the data collection was repeated, either with the nurse or therapist, or independently for the ST and UST trials, respectively.

### Data Collection Systems and Materials

For this study, demographic information was extracted by the clinical team from the clinical record and provided to the study team in a pseudoanonymized form. Gait data was collected using validated Physilog^®^5 (Gait Up), wearable inertial measurement units, to assess the spatiotemporal gait outcomes during the 10-MWT [35-37]. To keep the setup simple and easy for patients to use, only 2 sensors were used in this study. Each sensor was mounted on the top of the participant’s foot, specifically over the laces of their shoes (Figure S1 in Multimedia Appendix 1). The sensors had a sampling rate of 128 Hz and collected acceleration and orientation data. Gait data was subsequently analyzed using the Gait Analyser software (V3.1; Gait Up), a valid and reliable tool for stroke survivors [35,36]. Information about participant motivation was also collected using the Intrinsic Motivation Inventory as a standard pen-and-paper questionnaire that participants filled out in the nurses’ office when returning the sensors [38].

### Statistical Analysis

Descriptive statistics of all gait parameters were calculated to provide an overview of the data. For each assessment, speed, stride length, and cadence were calculated as the mean values of the left and right sensors. These specific gait parameters were chosen to provide a simplified overview of changes in temporal, spatial, and combined spatiotemporal parameters. Additionally, changes in these parameters are important to capture are they represent a more cautious gait in the elderly population and are known to change poststroke [39,40]. All statistical testing was conducted for each of the 3 gait parameters. Normality was determined using the Shapiro-Wilk test (significance level α=.05), while the variances were determined using an *F* test (α=.05). Corrections for multiple comparisons were done using the Holm-Bonferroni method.

#### Difference Between Repeated Measurements

In the first step, the measurements conducted on different days were compared via a paired *t* test to determine if there was any learning effect from measurement 1 to measurement 2 of the same assessment type. Additionally, using a 2-way random effect model and “single rater” unit, the intraclass correlation coefficient was computed to assess the agreement between the 2 measurements per assessment condition.

#### Sequence Effect

Next, to determine whether there was a sequence effect in the data, the difference in scores between the 2 measurements per assessment type in each sequence was calculated. For data found to be normally distributed and with equal variances, an independent samples *t* test was conducted between sequences (A vs B); for data found to be not normally distributed and with equal variances, a nonparametric Mann-Whitney *U* test was conducted. Significant differences between sequences A and B would suggest a sequence effect is present.

#### Supervised Versus Unsupervised Tests

The difference in scores between ST and UST measurements for each participant was determined using a student’s *t* test and a nonparametric Wilcoxon signed-rank test, depending on the outcomes of the normality testing.

#### Intrinsic Motivation Inventory

To determine if there were differences in motivation, the subsections of the Intrinsic Motivation Inventory were analyzed. Depending on the normality of the data, either a paired *t* test or a Wilcoxon signed-rank test was used to determine if there was a change in motivation from the first to second measurement day per assessment in each sequence. Additionally, a Student *t* test or a Wilcoxon signed-rank test was conducted to determine if there was a significant difference in motivation scores between assessment types (ie, ST vs UST).

### Ethical Considerations

This study was approved via waiver by the local ethics committee of the Canton of Lucerne, Switzerland (Req-2020-00995) and was carried out in accordance with the current version of the Declaration of Helsinki. The study protocol was explained both orally and in writing to eligible participants, and written informed consent was obtained before participation. Participants did not receive any compensation for their participation in this study, nor did they receive any direct benefit from their participation. To ensure privacy and confidentiality, all data were deidentified and securely stored in an encrypted database accessible only to authorized researchers. The wearable sensor data used for gait analysis were coded and stored separately from participant identifiers.

## Results

### Demographics

A total of 21 poststroke participants were recruited for this study (Table 1). All participants were able to complete all 4 days of testing. The majority of the participants were in the subacute phase and were largely community ambulators (Table 1) [41].

**Table 1:** Participant characteristics.

Characteristics				Values
Age in years, mean (SD)				63.91 (15.54)
**Sex, n (%)**				
	Female	10 (48)		
	Male	11 (52)		
**Paretic body side, n (%)**				
	Left	16 (76)		
	Right	5 (24)		
Months since stroke, mean (SD)			5.19 (10.73)	
Stroke onset 1-6 months, n (%)			18 (85.71)	
Stroke onset >6 months, n (%)			3 (14.29)	
Modified Rankin Scale, mean (SD)			1.95 (0.59)	
Household ambulators: <0.4 m/s, n (%)			1 (4.76)	
Limited community ambulators: 0.4-0.8 m/s, n (%)			4 (19.05)	
Community ambulators: >0.8 m/s, n (%)			16 (76.19)	
**Assistive device, n (%)**			2 (9.52)	
	Rollator	1 (4.76)		
	Dropped foot stimulator	1 (4.76)		
Foot orthoses, n (%)			3 (14.29)	

### Gait Outcomes

#### Difference Between Repeated Measurements

Shapiro-Wilk tests for normality showed that the results for speed, stride length, and cadence were normally distributed during each measurement, per assessment type and sequence (Table S1 in Multimedia Appendix 2). A paired samples *t* test subsequently determined that there were no statistically significant differences between the first and second measurements for each assessment type within each sequence (Table 2).

**Table 2:** Differences between repeated measurements.

Variable and sequence			Condition		*t* test^a^ (*df*)		*P* value^b^		Adjusted *P* value^c^		95% CI
**Speed**											
	A	ST^d^		0.492 (7)		0.64		>.99		–0.09 to 0.14	
	A	UST^e^		–0.064 (11)		0.95		>.99		–0.09 to 0.08	
	B	ST		–0.238 (11)		0.82		>.99		–0.08 to 0.07	
	B	UST		–5.10E–16 (7)		>.99		>.99		–0.06 to 0.06	
**Stride length**											
	A	ST		1.011 (7)		0.36		>.99		–0.05 to 0.12	
	A	UST		0.165 (11)		0.87		>.99		–0.06 to 0.07	
	B	ST		0.444 (11)		0.68		>.99		–0.05 to 0.08	
	B	UST		0.097 (7)		0.93		>.99		–0.12 to 0.13	
**Cadence**											
	A	ST		–0.322 (7)		0.76		>.99		–5.21 to 3.96	
	A	UST		–0.679 (11)		0.51		>.99		–5.41 to 2.86	
	B	ST		–0.132 (11)		0.90		>.99		–5.52 to 4.89	
	B	UST		0.353 (7)		0.73		>.99		–3.31 to 4.47	

^a^Paired samples *t* test.

^b^*P*≤.05.

^c^Holm-Bonferroni correction for multiple comparisons.

^d^ST: supervised test.

^e^UST: unsupervised test.

The intraclass correlation for speed found the ST (κ=0.93, adjusted *P<*.001) and UST (κ=0.93, adjusted *P<*.001) to both have excellent reliability across measurements. For stride length, the intraclass correlation was found to have excellent reliability for ST (κ=0.92, adjusted *P<*.001) and good reliability for UST (κ=0.88, adjusted *P<*.001). For cadence, both the ST (κ=0.91, adjusted *P<*.001) and UST (κ=0.95, adjusted *P<*.001) were found to have excellent reliability.

#### Sequence Effect

An independent samples *t* test found no sequence effect between sequences A and B for the normally distributed speed, cadence, and stride length (ST only; Tables S2 and S3 in Multimedia Appendix 2). A nonparametric Mann-Whitney *U* test equally found that there was no sequence effect between sequences A and B for the nonnormally distributed stride length (UST only; Table S2 and S4 in Multimedia Appendix 2). Before this, the results for the *F* test found no significant differences in variance across sequences (Table S5 in Multimedia Appendix 2).

#### Supervised Versus Unsupervised Tests

**Figure 3:**
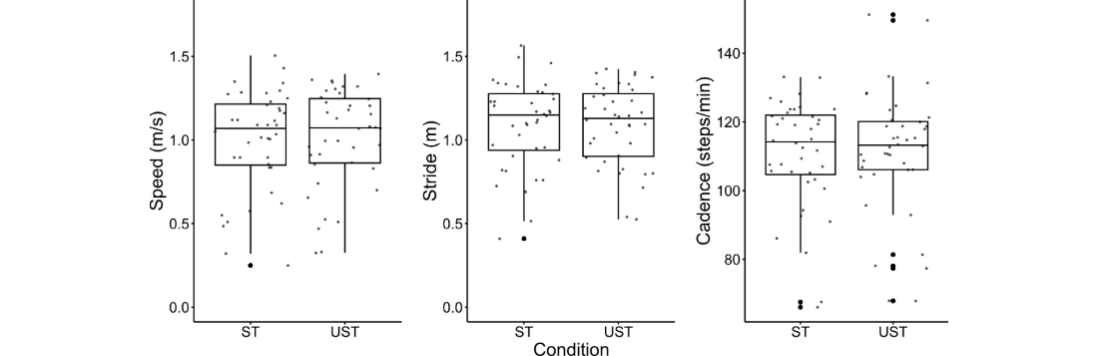
Distribution of data for speed (m/s), stride length (m), and cadence (steps/minute) during the ST and UST, with no significant differences found. ST, supervised test; UST, unsupervised test.

A paired-sample *t* test found that there was no significant difference between speed (t_39_=–0.735, adjusted *P*>.99, 95% CI –0.06 to 0.03) and cadence (t_39_=–0.501, adjusted *P*>.99, 95% CI –3.38 to 2.04) recorded when collecting data while supervised versus unsupervised (Figure 3). A pairwise Wilcoxon signed-rank test found that there was no statistically significant difference between the stride length (*z=*0.835, adjusted *P*>.99) collected during the ST versus UST. Additional results regarding the normality tests can be found in Table S6 in Multimedia Appendix 2.

The intraclass correlation coefficient was computed to assess the agreement between the supervised and unsupervised assessments of speed, stride length, and cadence (Table 3).

**Table 3:** Intraclass correlations between supervised and unsupervised assessments.

Variable	ICC value	*P* value^a^	*P* adjusted^b^	95% CI	Type of ICC^c^	Interpretation
Speed	0.90	<.001	<.001	0.81-0.94	ICC (A,1)	Good
Stride length	0.88	<.001	<.001	0.78-0.93	ICC (A,1)	Good
Cadence	0.88	<.001	<.001	0.79-0.94	ICC (C,1)	Good

^a^*P* value: 0.05.

^b^Holm-Bonferroni correction for multiple comparisons.

^c^Absolute agreement model for single measurement.

#### Bland-Altman Plots

**Figure 4:**
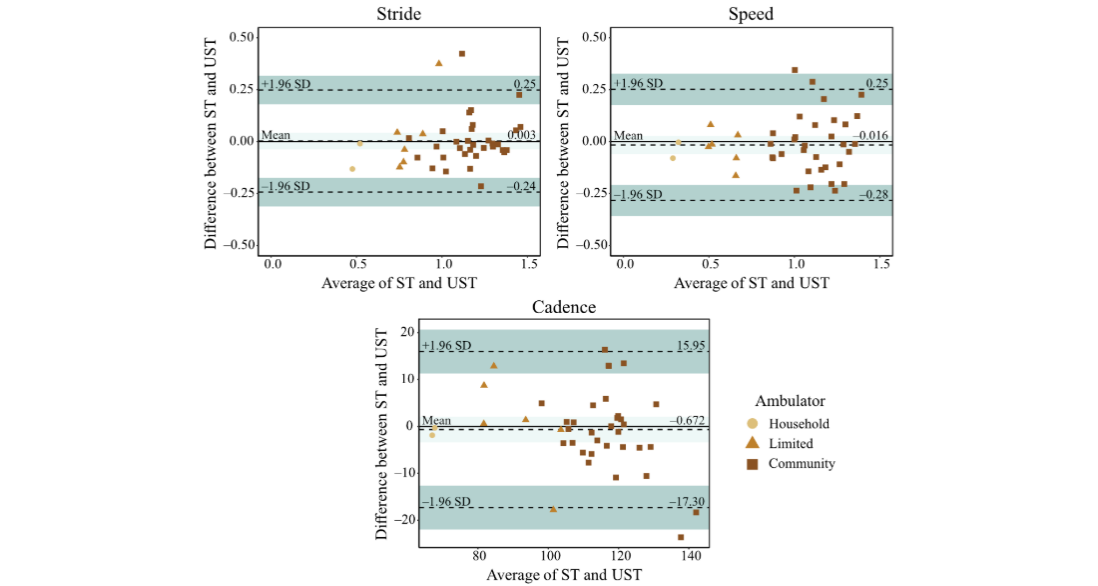
Results from the Bland-Altman analysis showing the various effect zones when examining the ST and UST for speed, stride length, and cadence. The darker bands represent the upper and lower limits of agreement, while the lighter band represents the bias zone. Data points are separated into ambulator types, namely household, limited, or community. ST: supervised test; UST: unsupervised test.

The effect of the assessment condition on the speed was found to result in a mean difference of –0.016 (SD 0.137) m/s during the UST compared with the ST. For stride length, the mean difference was found to be 0.003 (SD 0.125) m during the UST compared with the ST. For cadence, the mean difference was found to be –0.672 (8.481) steps per minute during the UST compared with the ST. Based on the Bland-Altman plots, no proportional bias was observed for any variable (Figure 4).

### Intrinsic Motivation Inventory Outcomes

The results of the Wilcoxon signed-rank test and paired-sample *t* test found that there are no significant differences in the motivation scores for the subscales of the Intrinsic Motivation Inventory between the ST and UST (Table 4).

**Table 4:** Comparison of the results of the Intrinsic Motivation Inventory, presented as its subscales, between the ST^a^ and UST^b^.

Subscale and condition		Mean (SD)	Score		*P* values		Adjusted *P* values^c^	
**Interest/enjoyment**				–0.194^d^		0.42		>.99
	ST	4.15 (1.62)						
	UST	4.00 (1.70)						
**Perceived competence**				–0.068^d^		0.47		>.99
	ST	5.14 (1.60)						
	UST	5.18 (1.44)						
**Effort/importance**				–0.088^d^		0.47		>.99
	ST	4.22 (0.83)						
	UST	4.28 (1.19)						
**Pressure/tension**				–0.435^e^		0.67		>.99
	ST	2.02 (1.55)						
	UST	2.10 (1.43)						

^a^ST: supervised test.

^b^UST: unsupervised test.

^c^Holm-Bonferroni correction for multiple comparisons.

^d^*z*-score.

^e^*t*-score.

Examining the difference between measurements 1 and 2, there were no significant differences for the interest/enjoyment, effort/importance, and pressure/tension subscales during both the ST and UST. Conversely, there was a significant difference in motivation scores for the Intrinsic Motivation Inventory subscale perceived competence during measurement 1 (mean 4.04, SD 1.58) versus measurement 2 (mean 5.17, SD 1.40) of the UST (*t*_7_=–2.979, *P=*.02, odds ratio 2.60, 95% CI –2.02 to –0.23) during sequence A. A significant difference between measurement 1 (mean 5.25, SD 1.33) and measurement 2 (mean 5.86, SD 1.13) was also found during sequence B (z*=*–1.716, *P=*.04). However, after applying the Holm-Bonferroni correction for multiple comparisons, the differences were no longer significant after adjustment (adjusted *P* values were .08 and .13, respectively). There were no differences between measurements in the perceived competence during the ST.

## Discussion

### Principal Findings

There is a considerable gap in our understanding of whether currently used standardized inpatient gait capacity assessments can be effectively performed in an unsupervised manner while maintaining the same levels of validity and replicability. Here, we specifically investigated the 10-MWT, a common measure of gait capacity, which is typically conducted under the supervision of a therapist to ensure accurate results. Our findings demonstrate that the execution mode of the 10-MWT, whether supervised by a health care professional or conducted autonomously by the participants themselves, did not result in significant differences in the measured gait parameters. These results suggest that patients are capable of accurately self-assessing their walking function without the need for direct supervision.

The main results of this study demonstrate that participants were able to maintain their execution ability without continuous guidance and feedback, suggesting that once an individual becomes familiar and comfortable with the assessment and related equipment, continuous supervision might not be necessary to preserve this ability. Moreover, these findings show the ability of stroke survivors to not only be able to carry out the test procedure, but also attach, connect, and start the required sensors with the help of a layperson after proper instruction. Getting patients involved in their own assessments may also promote greater flexibility and engagement in their rehabilitation process [42]. Moreover, therapists can efficiently monitor patients by leveraging reliable sensor systems for remote data collection, leading to more efficient treatment planning and timely interventions. This can not only reduce costs but also make rehabilitation more accessible and portable.

Enabling patients to conduct the assessments themselves could also have the added benefit of preventing experimenter-induced changes, which multiple studies have found to influence gait [32,43-45]. This is supported by work that found modifications in gait speed, cadence, step duration, and stride length, among others, in the presence of researchers [32,45-49]. Evidence also suggests that gait changes may even depend on the number of observers present [32]. However, the exact relationship between gait parameters and observation remains unclear due to contradictory results, which are thought to arise from differences in study design. While the studies mentioned here involved passive observers, another factor that could influence performance in the real world is motivation.

Motivation remains a clear confounder in the reliability of motor assessments, both for simple and complex tasks. For instance, Kuhl and Koch [50], modeled motor performance as a dual task or multiple tasks, with most nonmotor components being dependent on the environment, personality, and priming. Concerning the environment, sex, expectations, and the behavior of the experimenter have long been known to have an effect on motor performance [51,52]. In terms of personality, Strickland [53] reported the effects of the need-for-approval on motor outcomes in the 1960s. Regarding priming, a recent study demonstrated that priming with positive or negative content before a test resulted in distinct differences in simple motor capacity in healthy college students [54,55]. Based on this literature, we suspected that motivational scores would drop when no active experimenter was present to administer the test. However, in this study, we did not find this to be true. In fact, there were no reported differences in overall motivation between the supervised and unsupervised assessments. Before the correction for multiple comparisons, the perceived competence score during the unsupervised assessment was significantly lower during the first measurement compared with the second. Despite this finding no longer being significant after corrections were applied, this difference could still represent an important aspect to consider, as individuals may be less motivated to continue doing an assessment if they feel incompetent while doing it. This could be further examined in future work to determine whether this effect truly exists or not. Additionally, strategies to ensure that the highest level of understanding and skill is achieved during the unsupervised assessments should be anticipated.

### Limitations and Future Outlook

It should be noted that despite this study finding no changes in the motivation level between the 2 assessments, this finding is possibly biased, as participants were likely already highly motivated due to the study being voluntary. Despite this, there is currently no evidence to suggest that, when controlling for environment and setup, the presence or absence of an observer changes the motivation to complete the assessment.

To extend the generalizability of these findings to different environments, the next step would be to expand this work to the home environment. In doing so, future work would need to take into consideration the safety and feasibility of this setup outside of the clinical setting. While no participants in this study fell or were otherwise injured, a risk assessment should be conducted, including in other populations, to ensure patient safety. An additional aspect that may need to be considered for future work is cognitive ability, which was not examined in this study. As the majority of the participants were only mildly impaired, the applicability of the findings to patients with more severe impairment needs to be investigated. Finally, there are additional aspects, such as reduced costs and boosted self-efficacy, which were not considered here, that could be explored in future work as they could elucidate further benefits of autonomous assessments for clinics and patients.

### Conclusions

This validation study, using easy-to-use wearable sensors, has demonstrated that the 10-MWT, a standardized gait capacity assessment, can be performed reliably whether professionally supervised or conducted autonomously, without significant differences in the measured gait parameters. Additionally, there was no self-reported difference in motivation for completing the 10-MWT when conducted under supervised or unsupervised conditions. The findings of this study are important as they demonstrate the validity of the unsupervised versus supervised 10-MWT inside clinics. Furthermore, the results provide a solid foundation for investigating the validity of the 10-MWT outside of the clinic in combination with continuous monitoring. Both topics will play an increasingly important role as the frequency and granularity of clinical assessments increase and researchers begin to focus more on monitoring survivors’ rehabilitation progress and quality of life in the real-world setting.

## Appendix

Multimedia Appendix 1 Sensor placement on the top of the participant’s shoes.

Multimedia Appendix 2 Additional tables showing additional results per test type and sequence.

Multimedia Appendix 3 Full, preprocessed, dataset used for the data analyses outlined in the manuscript.

## Abbreviations

10-MWT: 10-Meter Walk Test
ST: supervised test
UST: unsupervised test

## Data Availability

All data generated or analyzed during this study are included in this published article and Multimedia Appendix 3.
