# Supplementary figures and images for: Toward Unsupervised Capacity Assessments for Gait in Neurorehabilitation: Validation Study
Source: J Med Internet Res. 2025 Mar 26;27:e66123. doi: 10.2196/66123 (PMC11982751; doi:10.2196/66123)

## Multimedia Appendix 1

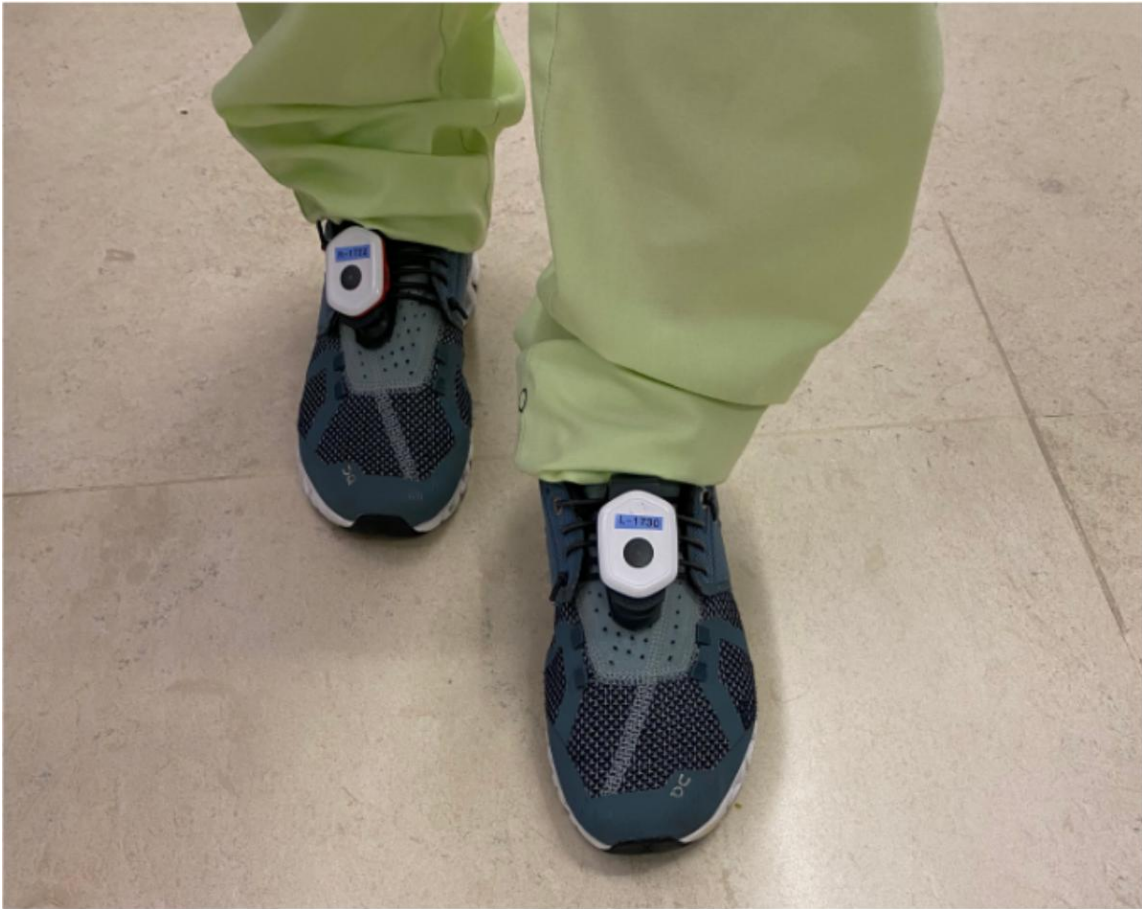

Figure S1. Sensor placement on the top of the participant's shoes.

Supplement: Multimedia Appendix 1 [file jmir_v27i1e66123_app1.pdf]
