# Supplementary material for: Toward Unsupervised Capacity Assessments for Gait in Neurorehabilitation: Validation Study
Source: J Med Internet Res. 2025 Mar 26;27:e66123. doi: 10.2196/66123 (PMC11982751; doi:10.2196/66123)
Supplement: Multimedia Appendix 2 [file jmir_v27i1e66123_app2.pdf]

Multimedia Appendix 2

Supplementary Tables:

Table S1: Normality results for each measurement, per test type and sequence.

Variable	Sequence	Condition	Measurement	Shapiro Wilk Test	
				W	*P* value
Speed	A	ST	M1	0.96	0.85
	A	ST	M2	0.89	0.25
	A	UST	M1	0.93	0.33
	A	UST	M2	0.92	0.31
	B	ST	M1	0.91	0.22
	B	ST	M2	0.97	0.94
	B	UST	M1	0.91	0.37
	B	UST	M2	0.89	0.24
Stride Length	A	ST	M1	0.97	0.89
	A	ST	M2	0.90	0.26
	A	UST	M1	0.96	0.79
	A	UST	M2	0.96	0.81
	B	ST	M1	0.95	0.57
	B	ST	M2	0.98	0.99
	B	UST	M1	0.94	0.58
	B	UST	M2	0.94	0.60
Cadence	A	ST	M1	0.86	0.11
	A	ST	M2	0.87	0.16
	A	UST	M1	0.91	0.20
	A	UST	M2	0.88	0.08
	B	ST	M1	0.94	0.51
	B	ST	M2	0.92	0.28
	B	UST	M1	0.91	0.38
	B	UST	M2	0.87	0.14

Table S2: Normality results for the differences between measurements during the ST and UST for both sequences A and B.

Variable	Sequence	Day	Condition	Shapiro-Wilk Test	
				W	*P* value
Speed	A	3-1	ST	0.93	0.52
	A	4-2	UST	0.97	0.90
	B	3-1	ST	0.92	0.30
	B	4-2	UST	0.86	0.11
Stride Length	A	3-1	ST	0.96	0.80
	A	4-2	UST	0.97	0.92
	B	3-1	ST	0.87	0.06
	B	4-2	UST	0.66	<0.001
Cadence	A	3-1	ST	0.96	0.78
	A	4-2	UST	0.92	0.31
	B	3-1	ST	0.95	0.60
	B	4-2	UST	0.88	0.20

Table S3: Sequence effect for normally distributed speed, cadence, and stride length data.

Variable	Condition	t^a	df	*P* value[a]	*P* adjusted[b]	(95% CI)
Speed	ST	–0.560	18	0.58	>.99	(–0.15 to 0.08)
	UST	0.047	18	0.96	>.99	(–0.12 to 0.11)
Stride Length	ST	–0.479	18	0.64	>.99	(–0.13 to 0.08)
Cadence	ST	0.094	18	0.93	>.99	(–6.65 to 7.28)
	UST	0.695	18	0.50	>.99	(–3.76 to 7.47)

[a] Independent samples t-test

[b] Holm-Bonferroni correction for multiple comparisons

Table S4: Sequence effect for non-normally distributed stride length data.

Variable	Condition	*z-score*[a]	*W*[a]	*P* value[a]	*P* adjusted[b]	(95% CI)
Stride Length	UST	–0.296	36	0.38	>.99	(–0.11 to 0.07)

[a] Mann-whitney U test

[b] Holm-Bonferroni correction for multiple comparisons

Table S5: Results of the F-test for variance between the differences of each condition across sequences.

Variable	Test	F_1-score	*df*	*P* value	*P* adjusted[a]	(95% CI)
Speed	ST	1.41	7	0.59	>.99	(0.37 to 6.62)
	UST	3.09	11	0.15	>.99	(0.66 to 11.60)
Stride Length	ST	0.93	7	0.95	>.99	(0.25 to 4.36)
	UST	0.52	11	0.32	>.99	(0.12 to 1.95)
Cadence	ST	0.45	7	0.30	>.99	(0.12 to 2.11)
	UST	1.95	11	0.38	>.99	(0.42 to 7.34)

[a] Holm-Bonferroni correction for multiple comparisons

Table S6: Normality results for the difference between the ST and UST across both sequences A and B.

Variable	Sequence	Condition	Shapiro-Wilk Test	
			W	*P* value
Speed	A & B	ST & UST	0.96	0.20
Stride Length	A & B	ST & UST	0.95	0.09
Cadence	A & B	ST & UST	0.86	<.001
